# From inflammation to invasion: neutrophils in cervical cancer pathogenesis

**DOI:** 10.1097/MS9.0000000000002679

**Published:** 2025-10-27

**Authors:** Emmanuel Ifeanyi Obeagu

**Affiliations:** Department of Biomedical and Laboratory Science, Africa University, Mutare, Zimbabwe

**Keywords:** cervical cancer, inflammation, metastasis, neutrophils, tumor microenvironment

## Abstract

Cervical cancer, predominantly caused by high-risk human papillomavirus (HPV) infections, continues to be a major health challenge globally. Recent research has highlighted the significant role of neutrophils, a type of white blood cell integral to the immune system, in the pathogenesis of cervical cancer. This review examines the dualistic role of neutrophils in cervical cancer, emphasizing their contribution to both inflammation and tumor progression. Understanding the intricate relationship between neutrophils and cervical cancer could unveil new therapeutic targets for better disease management. Neutrophils are key mediators of the immune response and inflammation. In the context of cervical cancer, these cells are recruited to the tumor microenvironment where they can adopt tumor-promoting phenotypes. Tumor-associated neutrophils (TANs) facilitate various processes that aid tumor growth and metastasis, such as producing reactive oxygen species (ROS) and proteases that induce DNA damage, releasing cytokines and chemokines that promote angiogenesis, and forming neutrophil extracellular traps (NETs) that enhance metastatic potential. Furthermore, TANs contribute to immune suppression by inhibiting the activity of cytotoxic T lymphocytes and natural killer cells, allowing cancer cells to evade immune surveillance. Given their pivotal role in cervical cancer progression, neutrophils represent a promising target for novel therapeutic strategies. Approaches such as inhibiting neutrophil recruitment to the tumor site, blocking NET formation, and modulating TAN phenotypes from pro-tumor to anti-tumor are being explored. These strategies aim to disrupt the supportive role of neutrophils in tumor development and progression, potentially leading to improved outcomes for patients with cervical cancer.

## Introduction

Cervical cancer is a significant global health problem, particularly affecting women in developing countries^[1]^. It is predominantly caused by persistent infection with high-risk human papillomaviruses (HPVs), especially HPV types 16 and 18. Despite advancements in screening and vaccination, cervical cancer remains the fourth most common cancer in women worldwide^[2]^. The pathogenesis of cervical cancer involves a complex interplay of viral oncogenes, host cellular responses, and the tumor micro environment (TME), where immune cells like neutrophils play crucial roles^[3–5]^. The immune system is a double-edged sword in cancer. It can recognize and destroy malignant cells, yet tumors often develop mechanisms to evade immune detection and destruction. In the TME, immune cells, including neutrophils, macrophages, and lymphocytes, are recruited and can either support anti-tumor immunity or facilitate tumor progression^[6]^. Neutrophils are the most abundant type of white blood cells and are essential components of the innate immune system^[7]^. They are the first responders to sites of infection and tissue damage, where they perform functions such as phagocytosis, degranulation, and the release of neutrophil extracellular traps (NETs). However, their role in cancer is complex, as they can be both pro-inflammatory and pro-tumorigenic, depending on the context. Chronic inflammation is a well-known risk factor for various cancers, including cervical cancer. Persistent HPV infection induces a chronic inflammatory response in the cervix, characterized by the recruitment of neutrophils^[8]^. These neutrophils release reactive oxygen species (ROS) and proteases, leading to DNA damage and promoting a pro-tumorigenic environment. The chronic presence of neutrophils in the TME thus plays a significant role in cervical carcinogenesis.

In the context of cancer, neutrophils can differentiate into tumor-associated neutrophils (TANs), which can exhibit either a pro-tumor (N2) or anti-tumor (N1) phenotype^[9]^. The N2 phenotype is typically associated with tumor progression, while the N1 phenotype can exert anti-tumor effects. In cervical cancer, the predominance of N2 TANs is linked to poor prognosis, as these cells enhance tumor growth, angiogenesis, and immune evasion. NETs are web-like structures composed of DNA and antimicrobial proteins that neutrophils release in response to infections^[10]^. While NETs play a protective role against pathogens, they have been implicated in cancer metastasis. In cervical cancer, NETs can trap circulating tumor cells (CTCs), facilitating their adhesion to distant organs and promoting the establishment of metastatic sites. This highlights the role of neutrophils not only in local tumor growth but also in the spread of cancer. Neutrophils secrete a variety of cytokines and chemokines that modulate the TME. For example, they produce vascular endothelial growth factor (VEGF), which promotes angiogenesis, and matrix metalloproteinases (MMPs), which degrade the extra cellular matrix (ECM) and facilitate tumor invasion^[11]^. These secreted factors create a microenvironment conducive to tumor growth and metastasis, underscoring the multifaceted role of neutrophils in cervical cancer. In addition to promoting tumor growth, neutrophils contribute to immune suppression in the TME. They release immunosuppressive factors such as arginase-1, which inhibits T-cell proliferation, and other molecules that impair the function of cytotoxic T lymphocytes (CTLs) and natural killer (NK) cells. This suppression of the anti-tumor immune response allows cancer cells to evade immune surveillance and proliferate unchecked. Given their significant role in the pathogenesis of cervical cancer, neutrophils represent a promising target for therapeutic intervention. Potential strategies include inhibiting neutrophil recruitment to the TME, blocking NET formation, and modulating the phenotype of TANs from pro-tumor to anti-tumor. These approaches aim to disrupt the supportive role of neutrophils in tumor development and enhance the efficacy of existing treatments.

**Highlights**

- Neutrophils exhibit dual roles in cervical cancer, promoting tumor progression through immune suppression and facilitating anti-tumor immunity.
- Understanding neutrophil-tumor interactions may guide targeted therapies.
- Neutrophil-related biomarkers could aid diagnosis and prognosis.
- Overcoming immunosuppressive neutrophil phenotypes presents therapeutic challenges.
- Future directions include personalized treatments and immunotherapy strategies targeting neutrophils for improved outcomes.

## Aim

The aim of this comprehensive review is to provide an in-depth exploration of the role of neutrophils in cervical cancer pathogenesis, with a focus on elucidating their diverse functions within the tumor microenvironment. By synthesizing current knowledge from preclinical and clinical studies, this review aims to:

1. Examine the dualistic nature of neutrophils in cervical cancer, encompassing both pro-tumorigenic and anti-tumorigenic activities.
2. Explore the mechanisms underlying neutrophil involvement in cervical cancer progression, including tumor promotion, immune suppression, and modulation of the tumor microenvironment.
3. Discuss the clinical implications of neutrophils in cervical cancer, including their potential as biomarkers for diagnosis, prognosis, and treatment response.
4. Identify challenges and future directions in neutrophil research in cervical cancer, with the goal of advancing our understanding and improving therapeutic strategies.

## Rationale

The rationale for conducting this comprehensive review on the role of neutrophils in cervical cancer lies in the increasing recognition of the complex interplay between the immune system and tumor progression. Neutrophils, traditionally viewed as the first line of defense against pathogens, have emerged as important players in the tumor microenvironment, exerting diverse functions that influence cancer development and progression. Cervical cancer represents a significant global health burden, and while advances have been made in understanding its pathogenesis and treatment, there remains a need for deeper insights into the tumor-immune interactions driving disease progression. Neutrophils have been implicated in various aspects of cervical cancer biology, including tumor promotion, immune evasion, and modulation of the inflammatory milieu within the tumor microenvironment. By conducting a comprehensive review of the current literature on neutrophils in cervical cancer, we aim to shed light on their multifaceted roles and their implications for disease prognosis and treatment outcomes. Understanding the mechanisms underlying neutrophil involvement in cervical cancer pathogenesis is critical for identifying novel therapeutic targets and developing more effective treatment strategies. Furthermore, by addressing the challenges and exploring future directions in neutrophil research in cervical cancer, we hope to pave the way for translational applications that can ultimately improve patient care and outcomes. By synthesizing existing knowledge and identifying gaps in our understanding, this review aims to stimulate further research in this important area of cancer immunology and contribute to the development of personalized and targeted approaches for the management of cervical cancer.

## Review methodology

### Literature search

A systematic literature search was conducted using electronic databases such as PubMed, Google Scholar, and Web of Science. The search terms included combinations of keywords related to neutrophils, cervical cancer, pathogenesis, mechanisms, clinical implications, challenges, and future directions.

### Inclusion criteria

Studies were included if they investigated the role of neutrophils in cervical cancer, either in preclinical models or clinical settings. Both original research articles and review articles were considered. No restrictions were placed on the publication date or language.

### Exclusion criteria

Studies that did not focus on neutrophils in the context of cervical cancer were excluded. Additionally, articles that did not provide relevant data or insights into the topic of interest were excluded.

### Data extraction

Relevant data from selected articles were extracted, including study objectives, methodologies, key findings, and conclusions. Emphasis was placed on synthesizing information related to neutrophil functions, mechanisms of action, clinical implications, challenges, and future directions.

### Data synthesis

Data from the selected articles were synthesized to provide a comprehensive overview of the role of neutrophils in cervical cancer pathogenesis. Common themes and patterns across studies were identified, and findings were organized into coherent sections to facilitate understanding and interpretation.

### Quality assessment

The quality of included studies was assessed based on study design, methodology, sample size, and relevance to the research objective. Studies with robust methodologies and clear research objectives were prioritized in the synthesis of findings.

## Limitations of the review

While the review provides a comprehensive examination of the role of neutrophils in cervical cancer pathogenesis, several limitations should be acknowledged:

### Limited scope of studies

#### Focus on specific aspects

- The review primarily focuses on specific aspects of neutrophil involvement, such as tumor promotion, immune suppression, and extracellular matrix remodeling. Other potential roles of neutrophils in cervical cancer, such as their interaction with other immune cells or their impact on cancer treatment responses, might not be covered in detail.

#### Variability in study design

- The included studies may vary in design, including differences in experimental models, sample sizes, and methodologies. This variability can impact the generalizability of the findings and make it challenging to draw consistent conclusions across studies.

### Data and evidence gaps

#### Incomplete data

- Some areas, such as the specific mechanisms through which neutrophils influence cervical cancer progression, might lack comprehensive data. For instance, while there is evidence linking neutrophils to inflammation and angiogenesis, detailed mechanistic insights might be limited.

#### Emerging research

- As research on neutrophils and cervical cancer is ongoing, new findings might emerge that were not included in the review. This could include recent discoveries related to neutrophil function, interactions with other cells, or novel therapeutic strategies.

### Clinical translation

#### Translational challenges

- The review highlights potential therapeutic targets and strategies but may not fully address the challenges of translating these findings into clinical practice. Issues such as the feasibility of targeting neutrophil functions in patients, potential side effects, and the effectiveness of proposed therapies in diverse patient populations might require further exploration.

#### Limited clinical evidence

- There may be limited clinical evidence directly linking neutrophil-targeted therapies to improved outcomes in cervical cancer patients. The review might focus more on preclinical and experimental data, with less emphasis on clinical trials and real-world applications.

### Heterogeneity of cervical cancer

#### Variability among subtypes

- Cervical cancer is a heterogeneous disease with different subtypes and stages. The role of neutrophils may vary across these subtypes and stages, which might not be fully addressed in the review. Specific interactions and impacts of neutrophils on different cervical cancer subtypes could be explored further.

#### Patient demographics

- Differences in patient demographics, such as age, ethnicity, and genetic background, might influence neutrophil function and its role in cervical cancer. The review may not fully account for these demographic variations and their potential impact on the findings.

### Methodological limitations

#### Selection bias

- The review might be subject to selection bias based on the studies included. Research with positive findings may be more likely to be published and included, potentially skewing the overall conclusions.

#### Quality of evidence

- The quality of evidence across studies may vary. Some studies might have limitations in study design, data collection, or analysis, which can affect the reliability of the conclusions drawn from the review.

## Overview of neutrophils

Neutrophils are the most abundant type of leukocytes (white blood cells) in the human body, constituting ~50–70% of the total circulating leukocytes^[12]^. They are a crucial component of the innate immune system and serve as the first line of defense against infections. Originating from hematopoietic stem cells in the bone marrow, neutrophils are short-lived but highly effective in their roles, with a lifespan of only a few hours to days once they enter the bloodstream. Neutrophils are primarily responsible for identifying, engulfing, and destroying pathogens through a process known as phagocytosis. They contain granules loaded with a variety of antimicrobial agents, including enzymes and reactive oxygen species (ROS), which are released to kill and digest invading microorganisms. Additionally, neutrophils can produce cytokines and chemokines that recruit and activate other immune cells, thus amplifying the immune response. In response to infection or tissue injury, neutrophils are rapidly recruited from the bloodstream to the site of inflammation. This process, known as chemotaxis, is mediated by signaling molecules such as chemokines (e.g. CXCL8/IL-8) and other inflammatory mediators^[13]^. Upon reaching the site of infection, neutrophils become activated and perform their antimicrobial functions, including degranulation, phagocytosis, and the formation of neutrophil extracellular traps (NETs). NETs are web-like structures composed of DNA, histones, and antimicrobial proteins that neutrophils release in response to various stimuli, including pathogens and inflammatory signals^[14]^. NETs trap and kill microbes extracellularly, preventing their spread. However, while NETs play a protective role in host defense, they can also contribute to tissue damage and chronic inflammation when not regulated properly, as seen in various inflammatory and auto immune diseases.

While neutrophils are essential for acute immune responses, their involvement in chronic inflammation can be detrimental. Persistent activation and recruitment of neutrophils can lead to continuous release of ROS and proteases, causing tissue damage and contributing to the development of chronic inflammatory conditions. This chronic inflammatory environment is a known risk factor for various cancers, including cervical cancer, where neutrophils play a significant role in disease progression.

In the context of cancer, neutrophils exhibit a dual role. On one hand, they can exert anti-tumor effects by attacking tumor cells and activating other immune cells. On the other hand, tumor-associated neutrophils (TANs) often adopt a pro-tumor phenotype, known as N2, which supports tumor growth, angiogenesis, and metastasis. The balance between these opposing roles is influenced by factors within the tumor microenvironment^[12]^. Neutrophils are not a homogeneous population; they exhibit plasticity and can differentiate into distinct phenotypes depending on the signals they receive from the environment. The N1 phenotype is associated with anti-tumor activity, characterized by the production of pro-inflammatory cytokines and ROS. In contrast, the N2 phenotype supports tumor progression through the secretion of anti-inflammatory cytokines, growth factors, and enzymes that remodel the extracellular matrix (ECM)^[13]^. In addition to promoting tumor growth, neutrophils can contribute to immune evasion by suppressing the activity of cytotoxic T lymphocytes (CTLs) and natural killer (NK) cells. They achieve this through the release of immunosuppressive molecules such as arginase-1, which depletes the essential amino acid arginine, inhibiting T-cell function. This immunosuppressive environment allows tumors to evade immune surveillance and grow unchecked. Given their significant role in cancer progression, targeting neutrophils offers a promising therapeutic strategy. Potential approaches include inhibiting the recruitment of neutrophils to the tumor site, blocking the formation of NETs, and reprogramming TANs from a pro-tumor (N2) to an anti-tumor (N1) phenotype. Such strategies could enhance the effectiveness of existing treatments and improve patient outcomes^[14]^.

## The role of the immune system in cancer

The immune system is a complex network of cells, tissues, and organs that work together to defend the body against infections and diseases. It consists of two main components: the innate immune system, which provides immediate, non-specific defense mechanisms, and the adaptive immune system, which provides long-lasting and specific protection against pathogens through the generation of memory cells. The intricate interplay between these components is essential for maintaining homeostasis and protecting the body from malignancies. The concept of immune surveillance suggests that the immune system continuously monitors and eliminates emerging tumor cells. This process involves the recognition of tumor-specific antigens presented by major histocompatibility complex (MHC) molecules on the surface of cancer cells. Cytotoxic T lymphocytes (CTLs) and natural killer (NK) cells are critical players in identifying and destroying these aberrant cells. Failure of immune surveillance can lead to the establishment and growth of tumors^[15]^. The tumor microenvironment (TME) is a dynamic and complex ecosystem comprising cancer cells, stromal cells, immune cells, and extracellular matrix components. The interactions within the TME play a pivotal role in cancer progression. Immune cells in the TME, such as macrophages, neutrophils, dendritic cells, and lymphocytes, can either suppress or promote tumor growth depending on their activation state and the signals they receive from the tumor and surrounding stroma^[10]^. Tumors have evolved several mechanisms to evade immune detection and destruction. These include the downregulation of antigen presentation molecules, secretion of immunosuppressive cytokines (e.g. transforming growth factor-beta (TGF-β), interleukin-10 (IL-10)), and the expression of immune checkpoint proteins such as programmed death-ligand 1 (PD-L1). These strategies create an immunosuppressive micro environment that impairs the function of anti-tumor immune cells, allowing the tumor to grow and metastasize.

Chronic inflammation is a well-established risk factor for cancer. Persistent inflammatory conditions can lead to an environment that supports tumor initiation and progression. Inflammatory cells, such as neutrophils and macrophages, release cytokines, chemokines, and growth factors that promote cellular proliferation, survival, angiogenesis, and metastasis. Furthermore, inflammation-induced oxidative stress and DNA damage contribute to genomic instability and carcinogenesis^[12]^. Macrophages are versatile immune cells that can adopt different phenotypes based on environmental cues. Tumor-associated macrophages (TAMs) are often polarized towards an M2 phenotype, which supports tissue remodeling, angiogenesis, and immunosuppression, thereby facilitating tumor growth and metastasis^[16]^. In contrast, M1 macrophages exhibit anti-tumor properties by producing pro-inflammatory cytokines and reactive oxygen species that can kill tumor cells. Neutrophils, another key component of the immune system, exhibit a dual role in cancer. They can contribute to anti-tumor immunity by directly killing tumor cells and facilitating the recruitment of other immune cells. However, tumor-associated neutrophils (TANs) often adopt a pro-tumor phenotype (N2), characterized by the production of factors that promote angiogenesis, tissue remodeling, and immunosuppression. This duality underscores the complex role of neutrophils in the TME^[15]^. One of the most significant advances in cancer immunotherapy is the development of immune check point inhibitors. These therapies block inhibitory pathways, such as the PD-1/PD-L1 axis, that tumors use to escape immune detection. By inhibiting these checkpoints, immune checkpoint inhibitors enhance the anti-tumor activity of CTLs and have shown remarkable efficacy in treating various cancers, including melanoma, lung cancer, and cervical cancer. Cancer vaccines aim to stimulate the immune system to recognize and attack tumor-specific antigens^[17]^. These vaccines can be prophylactic, like the HPV vaccine, or therapeutic, designed to treat existing cancers. Adoptive cell therapy, another promising approach, involves the infusion of ex-vivo-expanded tumor-infiltrating lymphocytes (TILs) or genetically engineered T cells (such as CAR-T cells) that are specific for tumor antigens. Both strategies aim to boost the body’s natural immune response against cancer.

## Neutrophils in chronic inflammation

Neutrophils are key components of the innate immune system, primarily known for their role in acute inflammation and rapid response to infection. They are the first immune cells to arrive at the site of infection or injury, where they perform functions such as phagocytosis, degranulation, and the formation of neutrophil extracellular traps (NETs). While these actions are crucial for controlling infections and initiating the healing process, the role of neutrophils in chronic inflammation presents a more complex and often detrimental picture^[8]^. The recruitment of neutrophils to sites of inflammation is a tightly regulated process involving the release of chemotactic signals such as chemokines (e.g. IL-8) and other inflammatory mediators. Once recruited, neutrophils become activated and release a variety of substances, including reactive oxygen species (ROS), proteolytic enzymes, and cytokines, which are essential for their antimicrobial functions. However, in chronic inflammatory conditions, this continuous recruitment and activation can lead to persistent tissue damage and contribute to disease progression^[6]^. Chronic inflammation is characterized by prolonged and persistent inflammatory responses that can last for months or even years. Unlike acute inflammation, which is self-limiting and resolves after the elimination of the initial insult, chronic inflammation results from a failure to resolve the inflammatory response. In this context, neutrophils play a central role in perpetuating inflammation through the continuous release of ROS and proteases. These molecules not only destroy pathogens but also damage surrounding tissues, leading to conditions such as fibrosis, tissue remodeling, and loss of function^[11]^. One of the significant consequences of chronic neutrophil activity is the induction of DNA damage. The ROS produced by neutrophils can cause oxidative damage to DNA, proteins, and lipids within cells. This damage can result in mutations and genomic instability, which are critical factors in the development of various cancers, including cervical cancer. Persistent neutrophil infiltration and the resulting DNA damage create a pro-tumorigenic environment, promoting the initiation and progression of malignancies^[12]^.

NETs are structures composed of decondensed chromatin and antimicrobial proteins that neutrophils release to trap and kill pathogens. While NETs play a protective role in acute infections, their persistent formation during chronic inflammation can have deleterious effects. NETs can contribute to tissue damage by inducing cell death and promoting thrombosis. In the context of cancer, NETs have been implicated in facilitating metastasis by trapping circulating tumor cells (CTCs) and aiding their dissemination to distant organs^[13]^. Several chronic inflammatory diseases are characterized by sustained neutrophil involvement. Conditions such as chronic obstructive pulmonary disease (COPD), rheumatoid arthritis, inflammatory bowel disease (IBD), and psoriasis all exhibit persistent neutrophil infiltration and activity^[18]^. In these diseases, neutrophils contribute to ongoing tissue damage and exacerbate disease symptoms through the continuous release of inflammatory mediators and degradative enzymes. The link between chronic inflammation and cancer is well-established, with neutrophils playing a pivotal role in this relationship. In chronic inflammatory environments, neutrophils promote cancer progression through various mechanisms, including the secretion of pro-tumorigenic cytokines, growth factors, and matrix metalloproteinases (MMPs)^[19]^. These factors support tumor growth, angiogenesis, and metastasis, highlighting the dual role of neutrophils in both protecting and promoting disease. Given the detrimental effects of neutrophils in chronic inflammation, therapeutic strategies aimed at modulating their activity are being explored. Approaches include the use of inhibitors to block neutrophil recruitment and activation, antioxidants to neutralize ROS, and agents that can disrupt NET formation. By targeting these pathways, it may be possible to reduce tissue damage and improve outcomes in chronic inflammatory diseases and cancer. Targeting neutrophils in chronic inflammation presents several potential benefits, such as reducing tissue damage, slowing disease progression, and improving patient quality of life. However, challenges remain, including the need to preserve the essential protective functions of neutrophils against infections and ensuring that therapies are specific enough to avoid widespread immunosuppression. Balancing these factors is crucial for the development of effective and safe treatments.

## Tumor-associated neutrophils (TANs)

Neutrophils, the most abundant type of white blood cells, are integral to the innate immune system and typically act as first responders to infection and tissue injury^[20]^. However, in the context of cancer, neutrophils can be co-opted by tumors to support their growth and spread. These neutrophils, known as tumor-associated neutrophils (TANs), exhibit a distinct phenotype and behavior compared to their counterparts in healthy tissue. TANs play a multifaceted role in the tumor microenvironment (TME), influencing cancer progression through a variety of mechanisms. The recruitment of neutrophils to the TME is mediated by chemokines and cytokines produced by both tumor cells and stromal cells^[21]^. Key chemokines involved in this process include CXCL1, CXCL2, and CXCL8 (IL-8), which bind to the CXCR1 and CXCR2 receptors on neutrophils, guiding them to the tumor site. Upon arrival, TANs are activated by various factors in the TME, such as granulocyte colony-stimulating factor (G-CSF) and tumor necrosis factor-alpha (TNF-α), which modulate their function and phenotype. Similar to macrophages, TANs can exhibit functional plasticity and differentiate into either pro-tumor (N2) or anti-tumor (N1) phenotypes, depending on the signals they receive from the TME^[9]^. The N1 phenotype is characterized by anti-tumorigenic properties, including the production of pro-inflammatory cytokines and reactive oxygen species (ROS), which can directly kill tumor cells. Conversely, the N2 phenotype promotes tumor growth and metastasis through the secretion of anti-inflammatory cytokines, growth factors, and matrix-degrading enzymes.

N2 TANs secrete vascular endothelial growth factor (VEGF) and other pro-angiogenic factors that promote the formation of new blood vessels, supplying the tumor with oxygen and nutrients necessary for its growth. N2 TANs produce immunosuppressive cytokines such as IL-10 and transforming growth factor-beta (TGF-β), which inhibit the activity of cytotoxic T lymphocytes (CTLs) and natural killer (NK) cells, allowing tumor cells to evade immune surveillance. By releasing matrix metalloproteinases (MMPs), N2 TANs degrade the extracellular matrix (ECM), facilitating tumor invasion and metastasis. N2 TANs can enhance the metastatic potential of cancer cells by producing chemokines and cytokines that support the survival and dissemination of circulating tumor cells (CTCs)^[21]^. Neutrophil extracellular traps (NETs) are web-like structures composed of DNA and antimicrobial proteins that neutrophils release in response to various stimuli. While NETs play a protective role in trapping and killing pathogens, their presence in the TME can have deleterious effects. NETs can promote cancer metastasis by trapping CTCs, aiding their adhesion to distant organs, and creating a supportive microenvironment for secondary tumor growth^[13]^. Chronic inflammation is a hallmark of many cancers and is closely associated with the recruitment and activation of TANs. Persistent inflammatory signals in the TME maintain TAN activation and support a pro-tumorigenic environment. The continuous presence of TANs and their inflammatory mediators can lead to sustained tissue damage, genomic instability, and further promotion of cancer progression^[9]^. Blocking chemokine receptors (e.g. CXCR2) or chemokine ligands (e.g., CXCL8) to prevent neutrophil infiltration into the TME^[22]^. Promoting the polarization of TANs towards the anti-tumor N1 phenotype through the use of cytokines or small molecules that alter the signaling pathways within TANs. Using DNase or other agents to degrade NETs and reduce their pro-metastatic effects. Targeting specific factors produced by TANs, such as VEGF or MMPs, to disrupt their pro-tumorigenic functions.

## Neutrophil extracellular traps (NETs) and metastasis

Neutrophil extracellular traps (NETs) are web-like structures composed of DNA, histones, and antimicrobial proteins that neutrophils release in response to infection, inflammation, or other stimuli. Initially identified as a mechanism for trapping and killing pathogens, NETs have since been implicated in various pathological processes, including cancer metastasis^[23]^. NETs are formed through a process called NETosis, which involves the release of chromatin and granule proteins from neutrophils^[24]^. This process can be triggered by various stimuli, including cytokines, microbial products, and tumor-derived factors. Upon activation, neutrophils undergo morphological changes and extrude their DNA, which becomes decorated with histones and antimicrobial proteins to form NETs. NETs can ensnare CTCs in the bloodstream, promoting their adhesion to endothelial cells and facilitating their extravasation into distant organs. NETs can stimulate the formation of blood clots (thrombosis) by activating platelets and the coagulation cascade. Tumor-associated thrombosis can provide a scaffold for CTCs and promote their survival and proliferation in the circulation. NETs can modulate the immune response by promoting the expansion of myeloid-derived suppressor cells (MDSCs) and inhibiting the activity of cytotoxic T lymphocytes (CTLs) and natural killer (NK) cells, thereby creating an immunosuppressive microenvironment that facilitates tumor progression and metastasis^[24]^. Studies have shown a correlation between elevated levels of circulating NETs and poor prognosis in cancer patients^[25]^,^[26]^. Increased NET formation has been observed in various cancer types, including breast cancer, lung cancer, and colorectal cancer, and is associated with metastatic spread and reduced survival. Furthermore, NETs have been detected within primary tumors and metastatic lesions, highlighting their involvement in the metastatic process. Blocking the molecular pathways involved in NET formation, such as the protein kinase C (PKC) pathway or the NADPH oxidase complex, to prevent excessive NET release^[27]^. Using recombinant DNase or other agents to degrade NETs and disrupt their pro-metastatic effects. Inhibiting specific proteins associated with NETs, such as histones or neutrophil elastase, to reduce their ability to promote metastasis. Stimulating the immune system to recognize and eliminate NET-producing neutrophils or targeting immunosuppressive cells recruited by NETs, such as MDSCs, to restore anti-tumor immunity.

## Cytokine and chemokine production by neutrophils

Cytokines and chemokines are small signaling molecules that play crucial roles in immune regulation, inflammation, and cell-to-cell communication^[28]^. While traditionally associated with immune cells such as macrophages and lymphocytes, emerging evidence indicates that neutrophils, traditionally considered short-lived effector cells, are also capable of producing a wide array of cytokines and chemokines. Neutrophils can secrete a variety of cytokines, including pro-inflammatory cytokines such as interleukin-1 (IL-1), interleukin-6 (IL-6), and tumor necrosis factor-alpha (TNF-α), as well as anti-inflammatory cytokines such as interleukin-10 (IL-10) and transforming growth factor-beta (TGF-β)^[29]^. The production of these cytokines by neutrophils can be induced by various stimuli, including microbial products, inflammatory mediators, and cellular interactions within the tumor microenvironment. Neutrophil-derived cytokines can contribute to the amplification and perpetuation of inflammatory responses. Pro-inflammatory cytokines such as IL-1, IL-6, and TNF-α promote the recruitment and activation of immune cells, vasodilation, and increased vascular permeability, leading to the influx of immune cells and inflammatory mediators to the site of inflammation^[30]^. While these responses are essential for host defense against pathogens, dysregulated production of pro-inflammatory cytokines can contribute to tissue damage and the pathogenesis of inflammatory diseases. In addition to pro-inflammatory cytokines, neutrophils can also produce anti-inflammatory cytokines such as IL-10 and TGF-β, which serve to limit and resolve inflammation. IL-10 inhibits the production of pro-inflammatory cytokines by other immune cells and suppresses the activation of antigen-presenting cells and T cells. TGF-β plays a crucial role in tissue repair and wound healing by promoting fibrosis and extracellular matrix deposition. However, excessive production of anti-inflammatory cytokines can impair host defense and promote immune evasion by tumors^[29]^.

Neutrophils can also secrete a variety of chemokines, which are small chemoattractant proteins that regulate the migration and activation of immune cells. Chemokines such as CXCL1, CXCL2, and CXCL8 (IL-8) are potent neutrophil chemoattractants and play a crucial role in recruiting neutrophils to sites of infection or inflammation. Additionally, neutrophils can produce chemokines such as CCL2 and CCL3, which attract other immune cells such as monocytes and T cells to the site of inflammation^[28]^. In the context of cancer, the production of cytokines and chemokines by neutrophils can influence tumor growth, progression, and metastasis. Pro-inflammatory cytokines and chemokines produced by neutrophils can promote angiogenesis, tumor cell proliferation, and invasion by activating signaling pathways involved in these processes. Conversely, anti-inflammatory cytokines and chemokines can suppress anti-tumor immune responses and create an immunosuppressive microenvironment that facilitates tumor evasion and metastasis^[27]^. Targeting cytokine and chemokine signaling pathways represents a promising therapeutic strategy for cancer treatment. Inhibitors of pro-inflammatory cytokines such as IL-1, IL-6, and TNF-α have shown efficacy in reducing inflammation and improving clinical outcomes in various inflammatory diseases and cancers. Similarly, inhibitors of chemokine receptors such as CXCR1/2 and CCR2/5 are being investigated as potential anti-cancer agents, with the aim of disrupting tumor-promoting inflammatory responses and inhibiting metastasis^[29]^.

## Immune suppression by neutrophils

While neutrophils are traditionally recognized for their role in the immediate defense against pathogens, emerging evidence suggests that they can also exert immunosuppressive effects, dampening immune responses and promoting immune evasion by tumors. Neutrophil-mediated immune suppression involves various mechanisms that modulate the activity of other immune cells and promote an immunosuppressive microenvironment conducive to tumor growth and progression. Neutrophils can directly inhibit T-cell responses through various mechanisms. One such mechanism involves the release of reactive oxygen species (ROS) and arginase-1, which deplete essential nutrients such as arginine and impair T-cell function. Neutrophils can also secrete immunosuppressive cytokines such as interleukin-10 (IL-10) and transforming growth factor-beta (TGF-β), which inhibit T-cell proliferation and cytokine production, further dampening anti-tumor immune responses^[31]^. Neutrophils can promote the differentiation and expansion of T regulatory cells (Tregs), a subset of T cells with immunosuppressive properties. Tregs play a critical role in maintaining immune tolerance and suppressing excessive immune responses. Neutrophils can stimulate Treg differentiation through the secretion of factors such as TGF-β and indoleamine 2,3-dioxygenase (IDO), creating an immunosuppressive microenvironment that favors tumor-immune evasion^[1]^.

Neutrophils can impair the function of antigen-presenting cells (APCs), such as dendritic cells (DCs), which are essential for initiating and regulating adaptive immune responses^[32]^. Neutrophils can inhibit DC maturation and antigen presentation through the release of factors such as prostaglandin E2 (PGE2) and reactive oxygen species (ROS), thereby limiting the activation of T cells and promoting immune tolerance. Natural killer (NK) cells are innate immune cells capable of recognizing and killing tumor cells without prior sensitization^[33]^. However, neutrophils can suppress NK cell activity through various mechanisms. Neutrophils can release factors such as TGF-β and vascular endothelial growth factor (VEGF), which impair NK cell function and promote their apoptosis. Additionally, neutrophils can physically interact with NK cells, forming neutrophil-NK cell aggregates that inhibit NK cell cytotoxicity. Neutrophils can promote the expansion and activation of myeloid-derived suppressor cells (MDSCs), a heterogeneous population of immature myeloid cells with potent immunosuppressive activity^[34]^. Neutrophils can induce the accumulation of MDSCs through the secretion of factors such as granulocyte colony-stimulating factor (G-CSF) and granulocyte-macrophage colony-stimulating factor (GM-CSF), creating an immunosuppressive environment that hinders anti-tumor immune responses. The immunosuppressive role of neutrophils has significant implications for cancer immunotherapy. Strategies aimed at targeting neutrophil-mediated immune suppression may enhance the efficacy of existing immunotherapies, such as immune checkpoint inhibitors and adoptive cell therapy. Potential approaches include inhibiting neutrophil recruitment or activation, blocking immunosuppressive factors produced by neutrophils, or promoting the differentiation and activity of anti-tumor immune cells.

## Cervical cancer pathogenesis

Cervical cancer is a multifactorial disease characterized by the progressive transformation of normal cervical epithelial cells into malignant tumors^[35]^. The pathogenesis of cervical cancer involves a complex interplay of genetic, environmental, and viral factors, culminating in dysregulated cell growth, invasion, and metastasis. Understanding the molecular mechanisms underlying cervical cancer pathogenesis is essential for the development of effective prevention strategies and targeted therapies. The primary etiological factor in the development of cervical cancer is infection with high-risk genotypes of human papillomavirus (HPV), particularly HPV types 16 and 18^[36]^. HPV infection is typically acquired through sexual contact and can lead to persistent infection of cervical epithelial cells. The viral oncoproteins E6 and E7 play a central role in HPV-associated carcinogenesis by disrupting key cellular pathways involved in cell cycle regulation, apoptosis, and DNA repair, ultimately promoting malignant transformation. Persistent infection with high-risk HPV can lead to the development of precancerous lesions known as cervical intraepithelial neoplasia (CIN). CIN lesions are characterized by dysplastic changes in the cervical epithelium, ranging from mild dysplasia (CIN1) to severe dysplasia/carcinoma in situ (CIN3). The progression from CIN to invasive cervical cancer is thought to occur through the accumulation of additional genetic and epigenetic alterations, resulting in the disruption of cellular homeostasis and the acquisition of invasive properties. Cervical cancer is associated with a variety of molecular alterations that contribute to tumor initiation, progression, and metastasis^[37]^. These alterations include mutations in tumor suppressor genes (e.g. TP53, PTEN) and oncogenes (e.g. PIK3CA, KRAS), as well as epigenetic changes such as DNA methylation and histone modifications. Dysregulation of signaling pathways such as the PI3K/AKT/mTOR pathway and the Wnt/β-catenin pathway is common in cervical cancer and promotes cell proliferation, survival, and invasion.

The TME plays a crucial role in cervical cancer pathogenesis by providing a supportive niche for tumor growth and progression^[38]^. The TME is composed of various cell types, including fibroblasts, immune cells, and endothelial cells, as well as extracellular matrix components and soluble factors such as cytokines and growth factors. Dysregulated interactions between tumor cells and the TME can promote tumor angiogenesis, immune evasion, and metastasis, contributing to disease aggressiveness and treatment resistance. Cervical cancer employs various immune evasion strategies to evade immune surveillance and promote tumor growth. HPV-induced tumors often exhibit immune evasion mechanisms such as downregulation of major histocompatibility complex (MHC) molecules, expression of immune checkpoint proteins (e.g., PD-L1), and recruitment of immunosuppressive cell populations such as regulatory T cells (Tregs) and myeloid-derived suppressor cells (MDSCs). These mechanisms create an immunosuppressive TME that hinders anti-tumor immune responses and promotes tumor progression^[39]^.

## The dual role of neutrophils in cervical cancer

Neutrophils, classically known for their role in the innate immune response against pathogens, exhibit a dual role in cervical cancer, contributing to both tumor progression and anti-tumor immunity. Understanding this dualistic nature of neutrophils in cervical cancer pathogenesis is crucial for developing effective therapeutic strategies and improving patient outcomes.

Neutrophils infiltrate the tumor microenvironment (TME) and differentiate into tumor-promoting phenotypes, characterized by the secretion of pro-inflammatory cytokines, growth factors, and matrix-degrading enzymes. These Tumor-Associated Neutrophils (TANs) create a pro-tumorigenic environment that supports tumor cell proliferation, invasion, and metastasis. Neutrophils release pro-angiogenic factors such as vascular endothelial growth factor (VEGF) and matrix metalloproteinases (MMPs), promoting the formation of new blood vessels within the tumor microenvironment. Enhanced angiogenesis facilitates tumor growth and metastasis by providing oxygen and nutrients to the growing tumor. Neutrophils can suppress anti-tumor immune responses by inhibiting the activity of cytotoxic T cells and natural killer cells, promoting the expansion of regulatory T cells (Tregs), and inducing the expression of immune checkpoint molecules such as programmed cell death-ligand 1 (PD-L1). These immunosuppressive effects create an environment that allows tumors to evade immune surveillance and escape destruction^[37–39]^.

Neutrophils can directly kill tumor cells through mechanisms such as antibody-dependent cellular cytotoxicity (ADCC), release of cytotoxic granules containing perforin and granzymes, and generation of neutrophil extracellular traps (NETs) that trap and kill tumor cells^[40]^. Neutrophils can stimulate anti-tumor immune responses by promoting the activation and recruitment of other immune cells, such as dendritic cells, T cells, and natural killer cells, to the tumor microenvironment. By enhancing immune cell infiltration and activation, neutrophils contribute to the initiation of adaptive immune responses against tumors. Neutrophils play a role in clearing apoptotic tumor cells and debris from the tumor microenvironment, preventing their accumulation and subsequent promotion of inflammation and tumor progression. The dual role of neutrophils in cervical cancer underscores the complexity of their interactions within the tumor microenvironment. Therapeutic strategies aimed at modulating neutrophil function must carefully balance the promotion of anti-tumor immunity while minimizing tumor-promoting effects. Potential approaches include targeting specific neutrophil subsets or phenotypes, manipulating signaling pathways involved in neutrophil recruitment and activation, and combining immunotherapies with conventional cancer treatments to enhance anti-tumor immune responses.

## Mechanisms of neutrophil involvement in cervical cancer

Neutrophils play multifaceted roles in the pathogenesis of cervical cancer, contributing to tumor progression through various mechanisms within the tumor microenvironment. Below are several key mechanisms through which neutrophils participate in cervical cancer:

### Tumor promotion and angiogenesis

Neutrophils are key players in the inflammatory response and have been shown to significantly impact tumor promotion and angiogenesis in cervical cancer. Neutrophils release proteolytic enzymes such as matrix metalloproteinases (MMPs), including MMP-9 and MMP-2. These enzymes degrade extracellular matrix (ECM) components, which facilitates tumor cell invasion and migration. The remodeling of the ECM by neutrophil-derived enzymes creates a more permissive environment for tumor growth and invasion, allowing cancer cells to penetrate surrounding tissues and establish secondary sites. Neutrophils secrete growth factors like vascular endothelial growth factor (VEGF) and fibroblast growth factor (FGF). VEGF stimulates endothelial cell proliferation and new blood vessel formation, while FGF promotes fibroblast growth and tissue repair. Neutrophils produce pro-inflammatory cytokines, such as IL-1β and TNF-α, which can enhance tumor cell proliferation and survival. These cytokines also recruit additional immune cells, perpetuating the inflammatory and tumor-promoting microenvironment. Neutrophils interact directly with tumor cells through adhesion molecules and surface receptors. These interactions can lead to the release of factors that promote tumor cell proliferation and resistance to apoptosis. Neutrophils can release cytokines and other factors that support tumor cell survival and proliferation, contributing to the overall progression of the cancer^[41]^. Neutrophils produce VEGF, a critical angiogenic factor that stimulates the formation of new blood vessels. The increase in blood vessel density provides tumors with a greater supply of oxygen and nutrients, supporting continued growth. By secreting MMPs, neutrophils contribute to the breakdown of the ECM, which is necessary for endothelial cells to migrate and form new blood vessels. Neutrophil Extracellular Traps (NETs), composed of DNA and antimicrobial proteins, can influence angiogenesis. NETs have been shown to promote the survival and proliferation of endothelial cells, aiding in new vessel formation. The presence of NETs creates a pro-inflammatory environment that can further stimulate angiogenesis and tumor growth. Neutrophils interact with endothelial cells through surface receptors and the secretion of angiogenic factors. This interaction can enhance endothelial cell proliferation and migration, leading to the formation of new blood vessels. Neutrophil-derived factors can directly stimulate endothelial cells, contributing to the expansion of the blood vessel network within the tumor. Elevated levels of neutrophils or neutrophil-derived markers in the blood or tumor microenvironment are often associated with poor prognosis in cancer patients. Monitoring these levels can provide insights into disease progression and treatment efficacy^[41]^.

### Immune suppression

In the complex tapestry of cancer progression, immune suppression plays a pivotal role, particularly in the context of cervical cancer. Cervical cancer, driven primarily by persistent infection with high-risk Human Papillomavirus (HPV), presents a classic example of how tumors can exploit immune suppression to thrive. As the tumor evolves, it begins to orchestrate a multifaceted strategy to subvert immune responses, creating an environment that fosters its growth and resistance to treatment. Within the cervical tumor microenvironment, various immune cells are recruited, including neutrophils, macrophages, and T lymphocytes. However, the very cells that are typically tasked with combating malignancy can become instrumental in supporting tumor growth due to immune suppression. Neutrophils, often seen as first responders to inflammation, may paradoxically promote tumor progression. Instead of merely combating cancer cells, they release factors that support angiogenesis and tissue remodeling, ultimately creating a more hospitable environment for tumor growth. Moreover, neutrophils can form extracellular traps that trap both cancer cells and immune cells, further complicating the immune response. Macrophages, particularly those adopting an M2-like phenotype, contribute significantly to immune suppression. These tumor-associated macrophages (TAMs) produce a range of cytokines and growth factors that not only support tumor growth but also suppress the activity of cytotoxic T cells and natural killer (NK) cells. By secreting IL-10 and TGF-β, TAMs inhibit the proliferation and activation of T cells, thereby dampening the anti-tumor immune response^[41,42]^.

Another crucial player in immune suppression is the regulatory T cell (Treg). Tregs are instrumental in maintaining immune tolerance and preventing autoimmunity, but in the context of cancer, they often become a double-edged sword. In cervical cancer, Tregs are frequently found in elevated numbers within the tumor microenvironment. These cells suppress the activation and proliferation of effector T cells, thereby blunting the immune response against tumor cells. The presence of Tregs can lead to a profound decrease in the efficacy of anti-tumor immunity. Cervical cancer cells also exploit immune checkpoint pathways to evade immune surveillance. Programmed death-ligand 1 (PD-L1) and programmed cell death protein 1 (PD-1) interactions are central to this immune evasion strategy. PD-L1 expressed on tumor cells binds to PD-1 receptors on T cells, leading to T-cell exhaustion and reduced anti-tumor activity. This mechanism allows the tumor to escape destruction by effectively turning off the immune system’s ability to recognize and target cancer cells. Chronic inflammation, a hallmark of persistent HPV infection, further exacerbates immune suppression. The inflammatory milieu generated by the continuous presence of HPV proteins can lead to the secretion of immunosuppressive cytokines and the recruitment of suppressive immune cells. This environment not only supports tumor growth but also creates a barrier to effective immune responses^[42]^.

### Tumor cell recruitment and invasion

Tumor cell recruitment and invasion are critical processes in cancer progression, enabling tumor cells to spread beyond their original site and establish secondary tumors. Tumor cells release a variety of signaling molecules, such as chemokines (e.g. CXCL12) and growth factors (e.g. VEGF), to recruit surrounding cells that support tumor growth and invasion. These factors can attract immune cells, fibroblasts, and endothelial cells, which contribute to the tumor’s progression. Tumor cells produce enzymes like matrix metalloproteinases (MMPs) that degrade Extracellular Matrix (ECM) components. This degradation not only facilitates tumor cell invasion but also alters the ECM to create a more supportive environment for tumor growth. Tumors often recruit macrophages, which can be polarized into a pro-tumorigenic M2 phenotype. Tumor-Associated Macrophages (TAMs) secrete cytokines and growth factors that support tumor cell proliferation and invasion. As previously discussed, neutrophils can also be recruited to the tumor site. While typically involved in combating pathogens, in the context of cancer, they can release factors that enhance tumor progression and invasion^[42]^. Tumors induce angiogenesis, the formation of new blood vessels, to supply the growing tumor with nutrients and oxygen. Tumor cells secrete angiogenic factors like VEGF, which recruit endothelial cells to form new blood vessels within the tumor. Tumor cells secrete Matrix Metalloproteinases (MMPs): that degrade various ECM components, such as collagen and fibronectin. This degradation creates pathways for tumor cells to invade surrounding tissues. In addition to MMPs, tumor cells may produce other proteases, like cathepsins, that contribute to ECM breakdown and tumor invasion. Tumor cells alter the expression of adhesion molecules, such as integrins and cadherins, which are crucial for their attachment to the ECM and surrounding cells. Changes in adhesion molecule expression can enhance the ability of tumor cells to migrate and invade. Tumor cells utilize various motility mechanisms, including ameboid and mesenchymal modes of movement, to navigate through the ECM and surrounding tissues. Actin remodeling and the formation of protrusions, such as lamellipodia and filopodia, facilitate this process. Tumor-associated fibroblasts and myofibroblasts secrete ECM components and growth factors that promote tumor invasion. They also interact directly with tumor cells, facilitating their migration and invasion. Immune cells in the tumor microenvironment can also contribute to invasion. For example, TAMs can release proteases and cytokines that support tumor cell invasion and disrupt normal tissue architecture. Epithelial-to-Mesenchymal Transition (EMT) is a biological process where epithelial cells lose their polarity and adhesion properties and acquire mesenchymal characteristics, such as increased motility. This transition is associated with enhanced invasive capabilities and is a key mechanism in cancer metastasis. Various signaling pathways, including TGF-β, Wnt/β-catenin, and Notch, regulate EMT. Tumor cells often activate these pathways to promote their invasive behavior^[42,43]^.

### Extracellular matrix remodeling

Extracellular matrix (ECM) remodeling is a fundamental process in tumor progression, impacting tumor growth, invasion, and metastasis. The ECM, a complex network of proteins and polysaccharides that provides structural and biochemical support to cells, is dynamically altered by tumor cells to facilitate their expansion and spread. The ECM is primarily composed of structural proteins such as collagen, elastin, and fibronectin. These proteins form a scaffold that supports tissue architecture and provides mechanical strength. Proteoglycans and Glycosaminoglycans (GAGs) including hyaluronic acid and heparan sulfate, contribute to ECM hydration, cell signaling, and tissue elasticity. The ECM provides a framework for cell adhesion and migration, influencing cell behavior and tissue integrity. ECM components interact with cell surface receptors to regulate signaling pathways that control cell proliferation, differentiation, and survival. Tumor cells produce MMPs, such as MMP-2, MMP-9, and MMP-14, which degrade ECM components like collagen and fibronectin. This degradation facilitates tumor cell invasion and migration. Cathepsins and serine proteases also play roles in ECM remodeling. These proteases contribute to the breakdown of ECM and activation of growth factors. Tumors often alter the composition and organization of collagen and fibronectin in the ECM. This alteration can lead to a more rigid or disorganized matrix, which can influence tumor cell behavior and enhance invasiveness. Increased production of hyaluronan, a GAG, can create a high-viscosity environment that promotes tumor growth and facilitates metastasis^[43]^.

Tumor-associated fibroblasts and myofibroblasts secrete ECM components and remodeling enzymes, contributing to the altered ECM environment. These cells play a crucial role in creating a tumor-supportive microenvironment. During angiogenesis, endothelial cells interact with the ECM to form new blood vessels. Tumor-induced ECM remodeling can influence the formation and function of these new vessels. ECM degradation creates physical pathways that tumor cells can use to invade surrounding tissues and enter the bloodstream or lymphatic system, leading to metastasis. Tumor cells modify their adhesion properties and use the remodeled ECM as a scaffold for migration. Changes in ECM stiffness and composition can enhance or inhibit cell motility. ECM remodeling facilitates the development of new blood vessels (angiogenesis), which supplies tumors with essential nutrients and oxygen, supporting continued growth. Proteolytic cleavage of ECM components can release sequestered growth factors, promoting tumor cell proliferation and survival. Tumor-induced ECM remodeling can attract immune cells to the tumor site. While some immune cells may aid in tumor control, others, such as tumor-associated macrophages, can support tumor progression by secreting pro-inflammatory cytokines and growth factors^[44]^.

### Tumor-associated inflammation

Tumor-associated inflammation is a key driver of cancer development and progression. Inflammation within the tumor microenvironment (TME) can have both supportive and destructive effects on tumor cells, influencing various aspects of tumor biology, from initiation to metastasis. Tumor cells and surrounding immune cells release cytokines such as tumor necrosis factor-alpha (TNF-α), interleukin-6 (IL-6), and interleukin-1β (IL-1β). These cytokines promote tumor cell proliferation, survival, and invasion while also recruiting additional immune cells to the tumor site. Chemokines like CCL2 and CXCL8 attract various immune cells, including macrophages and neutrophils, to the tumor. These chemokines contribute to the inflammatory milieu and can affect tumor progression. Vascular Endothelial Growth Factor (VEGF) is a key player in angiogenesis, promoting the formation of new blood vessels to supply the growing tumor with nutrients and oxygen. It is often upregulated in response to inflammatory signals. Transforming Growth Factor-beta (TGF-β) has a complex role in cancer. Initially, it can act as a tumor suppressor, but in established tumors, it often promotes immune suppression and epithelial-to-mesenchymal transition (EMT). Produced by inflammatory cells such as macrophages and neutrophils, Reactive Oxygen Species (ROS) can cause DNA damage, contributing to tumor initiation and progression. Nitric Oxide (NO), produced by inducible nitric oxide synthase (iNOS), can have both pro-tumor and anti-tumor effects, depending on its concentration and the context in which it is produced^[41–43]^.

Tumor-associated macrophages (TAMs) are often polarized into a pro-tumor M2 phenotype, which supports tumor growth by secreting growth factors, cytokines, and proteases. M2 TAMs promote angiogenesis, tissue remodeling, and immune suppression. The balance between M1 (pro-inflammatory) and M2 (anti-inflammatory) macrophages can influence tumor progression. M1 macrophages typically have anti-tumor effects, while M2 macrophages contribute to tumor growth and metastasis. Neutrophils recruited to the tumor site can release factors that promote angiogenesis, tissue remodeling, and immune suppression. They can also form neutrophil extracellular traps (NETs), which capture and potentially promote tumor cell dissemination. Regulatory T Cells (Tregs) often accumulate in the tumor microenvironment and suppress the activity of effector T cells, thereby allowing tumor cells to evade immune surveillance. High levels of Tregs are generally associated with poor prognosis. While cytotoxic T cells are capable of attacking tumor cells, their effectiveness can be diminished by the immunosuppressive environment created by other inflammatory cells and cytokines^[44]^. Chronic inflammation can lead to the accumulation of DNA damage due to ROS and other reactive molecules, increasing the likelihood of genetic mutations that drive cancer initiation. Inflammatory cytokines and growth factors stimulate tumor cell proliferation and survival, contributing to tumor growth. Inflammatory mediators such as VEGF promote the formation of new blood vessels, which is crucial for tumor growth and metastasis. Inflammation-induced production of proteases by immune cells and tumor cells remodels the extracellular matrix (ECM), facilitating tumor invasion and metastasis. The inflammatory environment can suppress anti-tumor immune responses through various mechanisms, including the activation of Tregs and the production of immune checkpoint molecules like PD-L1. Chronic inflammation can lead to the selection of tumor cells that are less recognizable by the immune system, contributing to immune evasion and resistance to immunotherapy^[6]^.

### Neutrophil extracellular traps (NETs)

Neutrophil extracellular traps (NETs) are web-like structures composed of DNA, histones, and antimicrobial proteins that are released by activated neutrophils. Initially described as a mechanism for trapping and killing pathogens, NETs have emerged as important players in various pathological conditions, including cancer. The process of NET formation, known as NETosis, involves the activation of neutrophils and the subsequent release of NETs. NETosis can occur through various pathways, including suicidal NETosis (where neutrophils undergo cell death to release NETs) and vital NETosis (where neutrophils release NETs while remaining alive). NETosis can be triggered by a range of stimuli, including microbial infections, pro-inflammatory cytokines, and immune complexes. In the context of cancer, tumor cells and associated factors can also induce NET formation. The core of NETs is composed of extracellular DNA, which serves as a scaffold for other components. Histones are nuclear proteins that bind DNA and are released into NETs, where they contribute to the antimicrobial activity and structural integrity of the traps. NETs contain various antimicrobial proteins and enzymes, such as neutrophil elastase, myeloperoxidase (MPO), and cathelicidins, which have antimicrobial and immune-modulating properties. NETs can trap and immobilize tumor cells, potentially influencing their growth and survival. This trapping may affect how tumor cells interact with the surrounding environment and other cells. NETs contribute to the inflammatory microenvironment of tumors by releasing pro-inflammatory cytokines and growth factors, which can promote tumor cell proliferation and survival^[6]^.

NETs have been shown to facilitate the spread of tumor cells to distant sites. Tumor cells can adhere to NETs and use them as a scaffold for dissemination through the bloodstream or lymphatic system. By trapping tumor cells within NETs, the immune system may have a reduced ability to recognize and eliminate these cells, potentially allowing them to survive and metastasize. NETs can influence ECM remodeling by interacting with ECM components and proteases. This interaction can affect tumor invasion and angiogenesis. NETs can promote angiogenesis by releasing growth factors and cytokines that stimulate new blood vessel formation, which supports tumor growth and metastasis. NETs can contribute to local immune suppression by affecting the activity of immune cells, such as T cells and macrophages. The components of NETs can modulate immune responses and reduce the effectiveness of anti-tumor immunity. The presence of NETs in the tumor microenvironment can influence the behavior of tumor-associated neutrophils (TANs), potentially altering their pro-tumor or anti-tumor functions. The presence of NET components, such as histones and MPO, in tumor tissues or blood samples can serve as diagnostic and prognostic markers. Elevated levels of NET markers may be associated with poor prognosis and increased metastatic potential. Strategies to inhibit NET formation or function could potentially reduce tumor progression and metastasis. For example, targeting NET-associated proteins or enzymes may help mitigate their pro-tumor effects. NETs can contribute to thrombosis, and antithrombotic agents that prevent NET formation or function may have potential benefits in cancer therapy by reducing tumor-associated thrombotic events. Combining therapies that target NETs with conventional treatments, such as chemotherapy or immunotherapy, may enhance overall treatment efficacy and improve patient outcomes^[45]^.

## Clinical implications in neutrophils in cervical cancer

Neutrophils play significant roles in the pathogenesis and progression of cervical cancer, with clinical implications that extend to diagnosis, prognosis, and treatment strategies. Understanding the clinical implications of neutrophils in cervical cancer is essential for optimizing patient management and improving outcomes. Here are some key clinical implications:

### Biomarkers for diagnosis and prognosis

Biomarkers are biological molecules found in blood, tissues, or other body fluids that can indicate the presence or progression of cancer. They play crucial roles in diagnosing cancer, determining prognosis, and guiding treatment decisions. Prostate-Specific Antigen (PSA) elevated levels are associated with prostate cancer. Carcinoembryonic Antigen (CEA) often elevated in colorectal and other cancers. CA-125 a marker for ovarian cancer. BRCA1 and BRCA2 mutations associated with breast and ovarian cancers. EGFR Mutations found in non-small cell lung cancer (NSCLC). MicroRNAs (miRNAs) are small RNA molecules that can be indicative of various cancers and are found in blood or tissues. Information about the size, extent, and aggressiveness of the tumor can help predict outcomes. TNM Staging System describes the extent of cancer in the body (Tumor size, Node involvement, Metastasis). Histological Grade **i**ndicates how abnormal the cancer cells look under a microscope. HER2/neu overexpression is linked to aggressive breast cancer and affects prognosis. KRAS Mutations: Associated with poorer prognosis in colorectal cancer. BCR-ABL Fusion Protein found in chronic myeloid leukemia (CML) and indicates disease progression. PD-L1 Expression high levels can predict response to immune checkpoint inhibitors. MSI-H (microsatellite instability**-**high**) i**ndicates potential response to immune checkpoint blockade in colorectal cancer^[45]^.

### Prognostic indicator

These indicators, which encompass a range of biological, clinical, and molecular factors, provide essential insights into the likely course and outcome of cancer. By integrating these indicators into clinical practice, healthcare providers can tailor treatment strategies, optimize patient care, and improve survival rates. Prognostic indicators are variables or measurements that help predict the likely progression of cancer and the patient’s overall survival chances. Unlike predictive biomarkers, which forecast a patient’s response to specific treatments, prognostic indicators provide a broader view of how the cancer may behave over time. They help answer crucial questions such as: How aggressive is the cancer? What is the likelihood of disease recurrence? What are the chances of survival? The stage of cancer, determined by the TNM system (Tumor size, Node involvement, Metastasis), is a fundamental prognostic indicator. For instance, early-stage cancers (Stage I and II) generally have a better prognosis compared to advanced stages (Stage III and IV) due to lower rates of metastasis and better treatment options. The grade of cancer, which reflects how abnormal the cancer cells appear under a microscope, provides insight into the tumor’s aggressiveness. Higher-grade tumors (Grade 3 or 4) tend to grow and spread more quickly than lower-grade tumors (Grade 1 or 2), impacting prognosis^[6]^.

Eastern Cooperative Oncology Group (ECOG) Scale assesses a patient’s overall health and ability to perform daily activities. A lower ECOG score (e.g. 0–1) indicates better functional status and is associated with a more favorable prognosis, while a higher score (e.g. 2–4) may suggest poorer health and a more guarded outlook. Specific genetic mutations in oncogenes, such as BRCA1 and BRCA2, are linked to various cancers, including breast and ovarian cancer. The presence of these mutations can influence treatment options and prognosis. Mutations in tumor suppressor genes, such as TP53, can lead to uncontrolled cell growth and are often associated with a poorer prognosis due to the aggressive nature of the cancer. Elevated levels of certain proteins, such as CA-125 in ovarian cancer or CEA in colorectal cancer, can indicate disease progression and affect prognosis. High levels of these markers often correlate with advanced disease and lower survival rates. Advanced genomic technologies allow for the analysis of gene expression patterns in tumors. For example, the Oncotype DX test provides a recurrence score that helps predict the likelihood of cancer recurrence in breast cancer patients, guiding treatment decisions. The presence and type of immune cells infiltrating the tumor, such as CD8+ cytotoxic T lymphocytes, can influence prognosis. High levels of immune cell infiltration often correlate with better outcomes due to enhanced immune surveillance and response. Markers of systemic inflammation, such as C-reactive protein (CRP) and interleukin-6 (IL-6), can provide prognostic information. Elevated levels of these markers are often associated with a worse prognosis and increased risk of metastasis. Prognostic indicators guide the selection of appropriate treatments. For example, patients with early-stage cancer and favorable prognostic factors may receive less aggressive treatment, while those with advanced-stage cancer and poor prognostic indicators may require more intensive therapy. Prognostic indicators are used to monitor disease progression and response to treatment. Changes in prognostic factors over time can provide insights into treatment efficacy and the need for adjustments in the management plan^[6,45]^.

### Therapeutic targets

Therapeutic targets are the molecules, pathways, or cells that play crucial roles in cancer progression and are therefore ideal candidates for intervention. By focusing on these targets, researchers and clinicians aim to develop treatments that are not only effective but also tailored to the unique characteristics of each patient’s cancer. Mutations in oncogenes, such as KRAS and MYC, can lead to uncontrolled cell growth. Targeting these mutated genes or their products with specific inhibitors can slow down or halt cancer progression. Mutations or loss of function in tumor suppressor genes like TP53 contribute to cancer development. Restoring the function of these genes or targeting their downstream effects presents promising therapeutic strategies. Many cancers exhibit the overexpression of proteins such as HER2 in breast cancer or EGFR in lung cancer. Monoclonal antibodies and small molecules targeting these proteins can block their activity and inhibit tumor growth. Proteins involved in critical signaling pathways, like the PI3K/AKT/mTOR pathway, are frequently altered in cancer. Inhibitors targeting these pathways can disrupt the signaling that promotes tumor survival and proliferation. Receptor Tyrosine Kinases (RTKs), such as EGFR and VEGFR, are often overactive in cancer cells. Targeted therapies that inhibit these receptors can reduce tumor growth and metastasis by blocking the signaling pathways they activate. Tumor cells often express unique antigens that are not present in normal cells. Therapeutic approaches, including cancer vaccines and chimeric antigen receptor (CAR) T-cell therapy, harness the immune system to target and destroy cancer cells displaying these antigens. Tumors require a blood supply to grow, and Vascular Endothelial Growth Factor (VEGF) is a key driver of angiogenesis. Anti-VEGF therapies, such as bevacizumab, inhibit new blood vessel formation, starving the tumor of necessary nutrients and oxygen. Tumor cells often evade immune detection by upregulating immune checkpoint proteins like PD-L1. Checkpoint inhibitors, such as pembrolizumab, block these interactions, enhancing the immune system’s ability to recognize and attack cancer cells. This innovative approach involves modifying a patient’s T cells to express chimeric antigen receptors that target cancer cells. These engineered T cells are then reintroduced into the patient’s body, where they seek out and destroy tumor cells. Novel strategies involve designing drugs that specifically target cancer cells while sparing healthy tissues. For example, antibody-drug conjugates link cytotoxic drugs to antibodies that bind to cancer cell-specific antigens, delivering the drug directly to the tumor. Combining therapies targeting multiple pathways or mechanisms can overcome resistance that arises from monotherapy. For instance, combining targeted inhibitors with immune checkpoint blockers can enhance overall treatment efficacy and reduce the likelihood of resistance. Tailoring treatments based on the specific genetic and molecular profile of a patient’s tumor allows for more precise targeting of therapeutic interventions. Genomic profiling can identify actionable mutations and guide the selection of targeted therapies^[6,45]^.

### Immunotherapy

Immunotherapy has emerged as one of the most exciting and transformative approaches in cancer treatment. By harnessing and enhancing the body’s immune system, this therapeutic strategy aims to target and eradicate cancer cells with precision. Unlike traditional treatments such as chemotherapy and radiation, which directly attack cancer cells, immunotherapy works by stimulating or restoring the immune system’s natural ability to recognize and destroy malignant cells. The immune system, a complex network of cells, tissues, and organs, is designed to protect the body from pathogens and abnormal cells. It comprises various components, including white blood cells (e.g. T cells, B cells, and macrophages), antibodies, and signaling molecules. In cancer, the immune system often fails to recognize and combat tumor cells due to several mechanisms: Tumor cells can produce signals that suppress immune responses or inhibit immune cell activity. Cancer cells may alter or hide their surface antigens, making it harder for immune cells to detect them. Immunotherapy seeks to overcome these challenges by enhancing the immune system’s ability to identify and destroy cancer cells. Some monoclonal antibodies block receptors on cancer cells that are involved in growth and survival. For example, trastuzumab (Herceptin) targets HER2-positive breast cancer cells. Others mark cancer cells for destruction by immune cells. For instance, rituximab targets CD20 on B cells in certain types of lymphoma. Programmed Cell Death Protein 1 (PD-1) Found on immune cells and interacts with PD-L1 on cancer cells to inhibit immune responses. Drugs like pembrolizumab (Keytruda) and nivolumab (Opdivo) block PD-1, enhancing immune activity against cancer. Cytotoxic T-Lymphocyte-Associated Protein 4 (CTLA-4) Inhibits T-cell activation. Drugs like ipilimumab (Yervoy) block CTLA-4, promoting stronger immune responses. Chimeric Antigen Receptor (CAR) T-cell therapy involves modifying a patient’s T cells to express a receptor that targets cancer cells. The process includes: CAR-T-cell therapy has shown remarkable success in certain blood cancers, such as acute lymphoblastic leukemia (ALL) and diffuse large B-cell lymphoma (DLBCL). Cancer vaccines are designed to stimulate the immune system to recognize and attack cancer cells. Oncolytic virus therapy involves using genetically modified viruses that selectively infect and destroy cancer cells while sparing normal cells. These viruses can also stimulate an immune response against the tumor. An example is talimogene laherparepvec (T-VEC), which is used for melanoma^[45,46]^.

### Potential for personalized medicine

Personalized medicine represents a revolutionary shift in how cancer is treated, moving from a one-size-fits-all approach to a tailored strategy based on an individual’s unique genetic, molecular, and clinical characteristics. This paradigm shift has the potential to enhance treatment efficacy, minimize side effects, and ultimately improve patient outcomes. Every cancer has a distinct set of genetic mutations that drive its development. By analyzing these mutations through techniques like next-generation sequencing (NGS), clinicians can identify specific alterations in a patient’s tumor that might be targeted by existing or experimental therapies. Certain patterns of mutations are associated with specific cancer types and can inform treatment decisions. For example, tumors with BRCA1 or BRCA2 mutations may respond to PARP inhibitors. Profiling the expression levels of various genes in a tumor can provide insights into its behavior and potential vulnerabilities. For instance, high expression of certain genes might indicate an aggressive tumor that requires more intensive treatment. Beyond genomics, analyzing the proteins and metabolites within a tumor can reveal additional therapeutic targets and biomarkers. Personalized medicine enables the identification of specific therapeutic targets based on a patient’s tumor profile. For instance, patients with HER2-positive breast cancer can benefit from HER2-targeted therapies like trastuzumab (Herceptin). By matching targeted therapies to the specific genetic and molecular profile of a patient’s tumor, treatments can be more effective and less likely to affect normal cells. Genetic and molecular profiling can help predict how a patient’s tumor will respond to specific drugs. This allows for the selection of therapies most likely to be effective and the avoidance of treatments that may be ineffective or harmful. Personalized medicine also aids in identifying mechanisms of drug resistance, enabling the development of second-line therapies or combination treatments to overcome resistance. Personalized medicine helps identify which patients are likely to benefit from immune checkpoint inhibitors based on biomarkers such as PD-L1 expression or tumor mutational burden. Engineering CAR-T cells to target specific antigens expressed by a patient’s tumor is a direct application of personalized medicine, leading to more effective and tailored immunotherapy treatments. Personalized vaccines can be created based on the unique antigens present in a patient’s tumor, offering a tailored approach to stimulate the immune system specifically against the patient’s cancer^[46]^. Table 1: Neutrophils in Cervical Cancer (provided by the author).

**Table 1:** Neutrophils in cervical cancer.

Aspect	Details
Role of neutrophils	Key players in inflammation, immune response, and tumor microenvironment in cervical cancer
Functions	Inflammation: Contribute to the inflammatory microenvironment.
	Angiogenesis: Promote blood vessel formation.
	Immune Suppression: Inhibit effective anti-tumor immunity.
	Tumor Invasion: Facilitate cancer cell migration and invasion.
	Extracellular Matrix Remodeling: Alter ECM to support tumor growth
Mechanisms of involvement	Inflammatory mediators: Release cytokines, chemokines, and proteases.
	NETs formation: Contribute to the formation of neutrophil extracellular traps (NETs) that influence tumor progression.
	Interaction with tumor cells: Directly interact with and influence tumor cells through cell surface receptors and secreted factors.
Tumor promotion	Inflammation and cytokine release: Neutrophils release cytokines (e.g. IL-6, IL-8) that promote tumor cell proliferation and survival.
	Angiogenesis: Secrete factors that stimulate blood vessel growth, aiding in tumor expansion.
Immune suppression	T-cell Inhibition: Neutrophils can suppress T-cell activation and function through the release of immunosuppressive cytokines and the expression of inhibitory molecules (e.g. PD-L1).
	Altered immune landscape: contribute to an immune-tolerant environment that supports tumor survival.
Tumor cell recruitment and invasion	Chemokine release: Neutrophils release chemokines (e.g. IL-8) that attract other immune cells and promote tumor cell migration.
	Matrix metalloproteinases (MMPs): Secrete MMPs that degrade the extracellular matrix, facilitating cancer cell invasion.
Extracellular matrix remodeling	Matrix degradation: Neutrophils produce enzymes that remodel the ECM, making it more conducive to tumor growth and metastasis.
	ECM components: Alter the composition of ECM components, affecting tumor cell adhesion and migration
Tumor-associated inflammation	Chronic inflammation: Persistent inflammation driven by neutrophils can lead to DNA damage and tumor promotion.
	Inflammatory microenvironment: The inflammatory environment supports tumor progression and resistance to therapy.
Neutrophil extracellular traps (NETs)	NETs formation: NETs consist of DNA and antimicrobial proteins that trap and kill pathogens but can also contribute to tumor growth by promoting a pro-inflammatory environment.
	Cancer cell interactions: NETs can interact with and modify tumor cells and the extracellular matrix, aiding in cancer progression
Biomarkers for diagnosis and prognosis	Neutrophil count and ratio: Elevated neutrophil count or high neutrophil-to-lymphocyte ratio (NLR) can indicate poor prognosis.
	NETs markers: Presence of NETs-related markers can serve as indicators of tumor progression and response to therapy
Prognostic indicators	Inflammatory markers: High levels of inflammatory cytokines and NETs can be associated with aggressive disease and poor outcomes.
	Neutrophil-Based Scores: Clinical scores incorporating neutrophil counts and related biomarkers can predict patient prognosis.
Therapeutic targets	Targeting neutrophils: Strategies to inhibit neutrophil recruitment, function, or NETs formation.
	Anti-inflammatory agents: Use of drugs that reduce inflammation or neutralize cytokines involved in tumor promotion
Immunotherapy	Enhancing immune response: Developing therapies that enhance the anti-tumor immune response while managing neutrophil-driven immune suppression.
	Combination approaches: Integrating immunotherapy with strategies to modulate neutrophil function and improve treatment efficacy

ECM, extracellular matrix.

## Challenges

Addressing the challenges in understanding the role of neutrophils in cervical cancer is essential for advancing research and improving patient outcomes. Neutrophils exhibit diverse functions within the tumor microenvironment, including both pro-tumorigenic and anti-tumorigenic activities^[47]^. Understanding the context-dependent nature of neutrophil function in cervical cancer is challenging but crucial for developing effective therapeutic strategies. Neutrophils are a heterogeneous population with distinct subsets that may have opposing roles in cancer progression. Identifying and characterizing specific neutrophil subpopulations in cervical cancer and elucidating their functional significance represent significant challenges. Neutrophils interact dynamically with other immune cells, such as T cells, macrophages, and dendritic cells, within the tumor microenvironment^[48]^. Deciphering the complex interplay between neutrophils and other immune cell populations in cervical cancer is challenging but essential for understanding immune regulation and tumor progression. There is a need for reliable biomarkers that reflect the functional state of neutrophils and their prognostic or predictive value in cervical cancer. Developing robust biomarkers for neutrophil activity in cervical cancer patients remains a challenge but holds promise for guiding treatment decisions and monitoring treatment responses. Translating preclinical findings on neutrophils in cervical cancer into clinical applications faces several challenges, including validating biomarkers, developing targeted therapies, and implementing personalized treatment approaches. Overcoming translational barriers is essential for translating basic research discoveries into clinically meaningful interventions. Neutrophils may contribute to therapeutic resistance in cervical cancer through various mechanisms, including immune evasion and promotion of angiogenesis^[49]^. Overcoming therapeutic resistance associated with neutrophil involvement poses a significant challenge in improving treatment outcomes for cervical cancer patients. There is a paucity of clinical data on the role of neutrophils in cervical cancer and their implications for patient prognosis and treatment response. Generating robust clinical evidence through well-designed studies is essential for validating the clinical relevance of neutrophil-related findings and guiding clinical practice. Addressing the challenges associated with neutrophils in cervical cancer requires interdisciplinary collaboration between researchers, clinicians, and industry partners. Collaborative efforts are essential for advancing our understanding of neutrophil biology, developing novel therapeutic strategies, and translating research findings into clinical practice.

## Future directions

Exploring future directions in the study of neutrophils in cervical cancer holds promise for advancing our understanding of tumor-immune interactions and improving therapeutic outcomes. Developing targeted therapies that specifically modulate neutrophil function within the tumor microenvironment represents a promising avenue for cervical cancer treatment^[50]^. Strategies targeting neutrophil recruitment, activation, or immunosuppressive functions may enhance anti-tumor immunity and improve treatment responses. Investigating the potential of immunotherapy strategies, such as immune checkpoint inhibitors or chimeric antigen receptor (CAR) T-cell therapy, to harness neutrophil-mediated anti-tumor immune responses in cervical cancer is an exciting future direction^[51]^. Enhancing the efficacy of immunotherapy by modulating neutrophil-tumor interactions could lead to improved patient outcomes. Identifying reliable biomarkers that reflect neutrophil activity and predict treatment responses in cervical cancer patients is critical for personalized medicine approaches. Future research should focus on validating neutrophil-related biomarkers and incorporating them into clinical practice for prognostication and treatment selection.

Exploring combination therapies that target both tumor cells and the tumor microenvironment, including neutrophils, holds promise for overcoming therapeutic resistance and improving treatment outcomes in cervical cancer. Combining conventional therapies with immunotherapy or targeted agents that modulate neutrophil function may synergistically enhance anti-tumor effects. Utilizing single-cell analysis techniques, such as single-cell RNA sequencing and mass cytometry, to dissect the heterogeneity of neutrophil populations in cervical cancer tissues could provide valuable insights into their functional diversity and clinical relevance^[52]^. Understanding the phenotypic and functional heterogeneity of neutrophils may guide the development of precision therapies. Developing advanced preclinical models, such as patient-derived xenografts (PDX) or organoid cultures, that recapitulate the complex tumor-immune interactions in cervical cancer will facilitate the evaluation of novel therapeutic strategies targeting neutrophils^[53]^. Preclinical models that faithfully mimic the tumor microenvironment can accelerate the translation of research findings into clinical applications. Conducting well-designed clinical trials to evaluate the safety and efficacy of neutrophil-targeted therapies in cervical cancer patients is essential for translating basic research discoveries into clinical practice. Future clinical trials should incorporate biomarker-driven patient selection and explore combination therapies to maximize treatment efficacy. Performing longitudinal studies to monitor changes in neutrophil phenotype and function throughout the course of cervical cancer treatment and disease progression could provide valuable insights into treatment responses and disease outcomes. Longitudinal profiling of neutrophil-related biomarkers may help identify early predictors of treatment response or resistance.

## Conclusion

The role of neutrophils in cervical cancer is a multifaceted and dynamic journey, one that places them at the crossroads of both tumor progression and immune defense. Their dualistic nature, exhibiting both pro-tumorigenic and anti-tumorigenic effects, reflects the complexity of their interactions within the tumor microenvironment. Neutrophils, classically recognized as the first responders to microbial threats, emerge as central players in the evolving narrative of cervical cancer. While they can promote immunosuppression, angiogenesis, and tumor invasion, they are equally capable of mounting cytotoxic responses, supporting anti-tumor immune actions, and restraining tumor growth. The precise role of neutrophils in cervical cancer depends on a constellation of factors, including the stage of the disease, the specific immune landscape, and the ever-changing microenvironment. This context dependency has profound clinical implications, extending from prognostic markers and predictive biomarkers to personalized treatment plans and therapeutic strategies.

## Ethical approval

Not applicable as this a review.

## Consent

Not applicable as this a review.

## Source of funding

No funding was received for writing this review paper.

## Author contributions

E.I.O. performed the following roles: conceptualisation, methodology, supervision, draft writing, editing and approval before submission

## Conflicts of interest disclosure

The author declares no conflicts of interest.

## Research registration unique identifying number (UIN)

Not applicable as this a review.

## Guarantor

The guarantor is Emmanuel Ifeanyi Obeagu.

## Data availability statement

Not applicable as this a review.

## Provenance and peer review

It is not invited.
